# Telecommuting and COVID-19: how has the pandemic changed workers’ perception on physical and mental health?

**DOI:** 10.47626/1679-4435-2023-856

**Published:** 2023-04-18

**Authors:** Omar Domínguez-Amorocho, Luz Mery Contreras-Ramos, María Fernanda Domínguez-Amorocho

**Affiliations:** 1 Facultad de Ciencias Médicas y de la Salud, Universidad de Santander (UDES), Bucaramanga, Santander, Colombia; 2 Facultad de Enfermería, Universidad Cooperativa de Colombia, Bucaramanga, Santander, Colombia; 3 Escuela de Ciencias Agrícolas, Pecuarias y del Medio Ambiente, Universidad Nacional Abierta y a Distancia (UNAD), Bucaramanga, Santander, Colombia

**Keywords:** COVID-19, teleworking, mental health, physical health, occupational medicine, COVID-19, teletrabajo, salud mental, salud física, medicina del trabajo

## Abstract

Due to the current pandemic situation, work from home, or telecommuting, has been implemented as part of public health measures to prevent the spread of SARS-CoV-2. Although this measure was introduced rapidly, it is likely to remain in effect for some time to prevent further outbreaks of COVID-19. Despite being few, various studies have addressed the relationship between telecommuting and workers’ health in the context of the current pandemic. Some aspects observed include fatigue, dietary changes, reduced levels of physical activity, and pain. Other conditions observed are associated with “techno-stress,” namely work overload, invasion of privacy, pace of information technology changes, decreased job autonomy, emotional exhaustion, and being constantly in electronic contact with work. Generally speaking, the COVID-19 pandemic has created a new environment for considering work and family life within the discussion on telecommuting. Likewise, a contextualized understanding of factors related to physical and mental well-being is essential to ensure positive impacts on workers. It is important to develop studies and discussions within organizations that allow knowing, analyzing and reformulating strategies and policies aimed at aspects such as changes in workers’ physical and mental well-being in the pandemic context and the way how occupational environments at home affect these components.

## Abstract (es)

Debido a la situación actual de la pandemia, el trabajo en casa, o *home office*, se ha implementado como parte de las medidas de salud pública para prevenir la propagación del SARS-CoV-2. Aunque esta medida se introdujo rápidamente, es probable que permanezca en vigor durante algún tiempo para evitar nuevos brotes de la COVID-19. A pesar de ser pocos, diversos estudios han abordado la relación entre el *home office* y la salud de los trabajadores en el contexto de la pandemia actual. Algunos aspectos observados incluyen fatiga, alteraciones en la dieta, reducción de la actividad física y dolor. Otras condiciones observadas se encuentran asociadas con el tecnoestrés, a saber: sobrecarga de trabajo, invasión de la privacidad, ritmo del cambio de las tecnologías de la información, menor autonomía laboral, agotamiento emocional y contacto electrónico constante con el trabajo. En términos generales, la pandemia de COVID-19 ha creado un nuevo entorno para considerar la vida laboral y familiar dentro de la discusión del *home office*. Así mismo, realizar una lectura en contexto de los factores que se relacionan con el bienestar físico y mental es fundamental para garantizar impactos positivos en los trabajadores. Es importante realizar estudios y discusiones al interior de las organizaciones que permitan conocer, analizar y reformular estrategias y políticas orientadas a aspectos como las alteraciones en el bienestar físico y mental de los trabajadores en el contexto de la pandemia y la forma en que los entornos ocupacionales en el hogar afectan estos componentes.

On March 11, 2020 the World Health Organization officially declares the COVID-19 pandemic, caused by the transmission of virus from the coronavirus family named SARS-CoV-2,^1^ leading to an unprecedented situation that exerted various economic and health impacts and generated prolonged periods of quarantine and social distancing as prophylactic strategies to reduce transmissivity.^2,3^ From that moment on, everyday life has experienced a radical change that has affected different aspects of life, particularly social dynamics, teaching and learning strategies, work processes and environment, among others. With regard to the latter aspect, the pandemic has caused the loss of thousands of jobs and, on the other hand, the “transformation” of homes into places of work, education, and leisure.^4,5^

Due to the current pandemic situation, working from home, or telecommuting, has been implemented as part of general public health measures to prevent the spread of SARS-CoV-2. Although this measure was introduced rapidly, it is likely to remain in effect for some time, and organizations will use it as a strategy to manage physical distancing requirements necessary to prevent further outbreaks of COVID-19.

The boom of information and communication technologies experienced over the last years has permeated work patterns. However, the context of teleworking and its general conditions, as acknowledged by the International Labor Organization (ILO), has existed since the 1970s, despite with a focus and extent much lower than that observed in the current context.^6^ A series of positive benefits have been associated with teleworking, such as improved family-work integration, reduction of fatigue, and improved productivity. Nonetheless, the blurring of physical and organizational boundaries between work and home has also brought important effects on workers’ physical and mental health, due to extended working hours, lack of or unclear work-home boundaries, and limited support from organizations or employers.^7,8^

During the COVID-19 pandemic, many workers were recommended for or simply transferred to teleworking or full-time telecommuting, which redefined the conventional concept of teleworking, which was restricted to certain types of job, on an occasional basis, or unique employees’ circumstances. Many companies believe that telecommuting will become increasingly common after the pandemic, since some organizations have conducted adaptations or set up remote work systems for their employees.^9^ Additionally, an additional challenge was posed by the possibility of decreasing operational costs for organizations with the reduction of administrative and rental costs.^9,10^

Some studies out of the pandemic context showed some advantages of telecommuting, since it allows for employees to choose to work when they are more productive (although it cannot be applied as a general rule), in addition to increasing productivity by reducing distractions that may be present in the work environment.^11^ Furthermore, workers can have greater control of environmental factors (e.g., lighting, temperature, humidity, air quality, noise, ergonomics, etc.), important aspects for workers’ physical and mental health.^12^ However, paradoxically, the pandemic context and structural and functional conditions of houses have a negative influence on the execution of telecommuting.

Several studies have addressed the effects of teleworking in different contexts, such as physical and mental health, social structures, economic impact, and productivity; nonetheless, available information on these events has not been studied or published in depth in the context of COVID-19 pandemic. With regard to the observed effects, some studies have shown a small, despite statistically significant, reduction in health self-perception, particularly those related to fatigue (including burnout, tiredness, or changes in energy levels), dietary changes, reduced levels of physical activity, and pain.^13-17^ Teleworkers do not have the opportunity of socialize with their colleagues and may present with physical movement limitations.^18^ Moreover, prolonged hours of screen exposure due to full-time work on the computer have also been associated with fatigue, tiredness, headache, and visual symptoms.^19^

It bears highlighting that most recent studies focused on the effects on mental health, considering environmental, organizational, physical, or psychosocial factors. Some aspects considered in these studies are associated with “techno-stress” (defined as work overload, invasion of privacy, and role ambiguity) and showed greater association with work overload, invasion of privacy, pace of information technology changes, decreased job autonomy, emotional exhaustion, and being constantly in electronic contact with work.^20-23^ Differences in organizational responses and support were found to contribute in increasing or mitigating negative health outcomes. Likewise, some studies support the important role of work environment (e.g., leadership, collegial support, work design) in employers’ health.^24,25^

A common problem in work-life boundaries is balancing work schedules with other family members.^26^ In some cases, workers, especially those who are parents, may choose to sacrifice their sleep hours and work nights or early mornings, as these are the only quiet hours when they can focus on work and avoid frequent interruptions.^27^ The continuous work-to-family conflict has also been related to emotional exhaustion.^23^

In addition to social and behavioral changes, telecommuting during the COVID-19 pandemic has also highlighted the need for physical space in their home office environment. It is worth emphasizing that not all workers have access to dedicated workstations at home, which may result in sharing their workstation with other family members, creating makeshift desks, or working at various places during the day,^28^ leading to important impacts on processes and ergonomic phenomena and on general workers’ quality of life.

The current pandemic situation has led to many sudden and unexpected changes in work practices that potentially create uncertainty to workers, which requires regular communication to ensure clarity regarding role expectations, clearly defined performance measures, appropriate workloads, and access to human resource support.^15,20^ Additionally, perceived and observed aspects of telecommuting related to workers’ physical and mental health, as well as elements of prevention and mitigation, should be considered as relevant aspects in defining and adapting policies to manage human resources in the context of the new and variable pandemic reality.^29,30^

Generally speaking, the COVID-19 pandemic has created a new environment for considering work and family life within the discussion on telecommuting. Likewise, a thorough understanding of factors related to physical and mental well-being is essential to ensure positive impacts on workers. It is important to develop studies and discussions within organizations that allow understanding, analyzing and reformulating strategies and policies aimed at aspects such as changes in workers’ physical and mental well-being in the pandemic context and the way how occupational environments at home affect these components.
